# A simulated annealing with graph-based search for the social-distancing problem in enclosed areas during pandemics

**DOI:** 10.1371/journal.pone.0318380

**Published:** 2025-02-11

**Authors:** Bayram Dundar

**Affiliations:** Department of Industrial Engineering, School of Engineering, Architecture, and Design, Bartin University, Bartin, Turkey; Aalto University, FINLAND

## Abstract

During the pandemic, decision-makers offered many preventive policies to reduce the negative effects of the pandemic. The social distance rule in enclosed areas was implemented by educational institutions in any countries. In this study, we deal with the problem of assigning students to seats by considering the social distancing constraint and with objective of maximizing the total distance among the students. This problem is found to be similar to the Maximum Diversity Problem (MDP) in the literature. We name this new problem as Maximum Diversity Social Distancing problem (MDPs). A simulated annealing algorithm framework for MDPs (SA-MDPs) is proposed to identify an optimal or near-optimal solution within a reasonable computational time. A greedy random-based algorithm is presented to determine efficiently an initial feasible solution. The new neighborhood search procedure based on graph theory is introduced, in which the dominated, dominating, and nondominated seats are determined based on social distance. The proposed SA-MDPs is evaluated on classrooms with varying capacities and benchmarked against an off-the-shelf optimization solver. The computational tests demonstrated that the SA-MDP model consistently provided either proven optimal solutions or superior best-known solutions compared to a commercial solver, all within a reasonable CPU time.

## Introduction

Due to the Covid-19 pandemic, many countries experienced a standstill in their economy, trade, education, and health sectors. As of October 2023, the United States has the highest number of confirmed Covid-19 deaths in the world, with a cumulative total of approximately 1.14 million deaths. Worldwide, the pandemic has claimed the lives of nearly 7 million people as reported by Mathieu et al. [1]. Almost all countries made school closures mandatory in April 2020 during the ongoing pandemic, according to the report. As time progressed, schools started to open up partially. Due to the pandemic, many countries worldwide have made significant decisions. The significant part of countries have implemented various measures and policies to combat the pandemic. One measure to prevent the spread of the virus is social distancing in closed areas and limiting the number of people to the predetermined capacity. According to Fong et al. [2], practicing physical distancing could be useful strategy to lessen the spread and effects of a pandemic. Courtemanche et al. [3] used an event-study model to predict how the number of cases would be affected without social distancing measurement. They estimated the number of cases would be more than ten times higher without social distancing. The mitigation methods to prevent the spread of the virus can alleviate the burden on the healthcare system. This will delay the peak of the epidemic, reduce its intensity, and spread cases over a longer period. Social distancing is implemented by educational institutions to reduce the risk of virus contamination by preventing close contact between people. It is crucial for the students to continue their education during the pandemic in terms of both their careers and keeping the wheels of the economy turning.

Social distance refers to the physical distance maintained between individuals who are not from the same family or group, particularly in enclosed areas during a pandemic. Viruses such as COVID-19 are typically transmitted to healthy individuals through droplets released into the air by infected people when they cough or talk. To reduce the risk of contamination and to maintain the workload of healthcare institutions at manageable levels during the pandemic, most countries implemented strict social distancing policies. The Centers for Disease Control and Prevention (CDC) [4] recommended a minimum social distance of 6 feet (1.8 m). However, different countries adopted varying social distancing measures. For instance, 45% of countries recommended a minimum distance of 1 meter, while 49% recommended greater distances [5]. Denmark implemented a 2-meter rule, Switzerland set the distance at 1.5 meters, and Turkey mandated a minimum of 1 meter along with a maximum capacity limit of 4 m^2^ per person in enclosed areas. A study conducted by Thu et al. [6] found that implementing social distancing during the pandemic’s peak in 10 countries significantly reduced infection rates. For example, the number of reported cases decreased by 71% in China, 48% in France, and 25.9% in the UK after social distancing measures were enforced. In addition to social distancing, wearing face masks and washing hands for at least 20 seconds were among the precautions recommended by the CDC. A study by Kwon et al. [7], which involved approximately 200,000 participants, reported a 31% lower risk of transmission in communities practicing social distancing. Similarly, the use of masks reduced the risk of contamination by 62%.

Placing the students to seats and considering the social distance constraint can be an intractable process for decision-makers. Dundar and Karakose [8] proposed mathematical models and heuristic algorithms to determine the maximum number of students that can be fitted in a classroom while satisfying a range of social distance options. However, maximizing the total distance among the students while assigning the students to seats is a more promising way to reduce the risk of virus transmission as [9] showed that the probability of virus contamination decreases with an increase in distance. Depending on the number of students in the classroom, maintaining the minimum social distance between students and assigning them to seats as far away from each other as possible decreases the risk of virus transmission, as highlighted in the study by Sun and Zhai [9]. The authors discussed the effectiveness of total social distancing in preventing the spread of the pandemic. They proposed a distance index *P*_*d*_, which indicates that the probability of virus transmission decreases as the physical distance between individuals increases. They concluded that a social distance of 1.6–3.0 m is considered a safe range for reducing contamination risks. During the pandemic, different countries implemented varying social distancing measures. While some countries adopted a minimum distance of 1 m, others enforced a distance of 3 m. In this study, the total distance is maximized by assigning students to seats under the constraint of maintaining a minimum social distance. This approach aligns with the findings of Sun and Zhai [9], which suggest that when a group of students is assigned to a confined space with a capacity below normal levels, maximizing the total distance between them effectively reduces the overall risk of virus transmission.

In this study, we aim to assign the students to available seats in such a way that the total distance among students is maximized without exceeding the capacity constraint. Maximizing the minimum distance between students while satisfying the minimum needed social distancing was considered by Dundar and Karakose [8]. The study also determined the maximum number of students that can be assigned to classrooms according to social distance scenarios. The minimum distance between students was maximized with a mathematical model. However, in this study, we maximize the total distance to mitigate the risk of virus contamination. A literature review shows that this problem resembles the maximum diversity problem from the aspect of maximizing the distance while picking the optimal sub-points in a given set. With this classification, in this study, the MDP social distancing problem formulation is employed. As the number of seats and students to be seated in the classroom increases the MDPs problem becomes intractable to solve in an efficient computation time. MDP is classified as an NP-hard problem. Heuristic and metaheuristic algorithms can be utilized to obtain an optimum or near-optimal result within an efficient time frame. We present a simulated annealing algorithm framework solution approach(SA-MDPs) for solving MDPs that allows decision-makers to assign students to seats within a reasonable time. The simulated annealing framework constitutes the neighborhood search and the initial feasible solution procedure, which are problem specific, namely, it can not be applied to all combinatorial problems. In the SA-MDPs, the proposed greedy-random-based initialization algorithm provides a feasible initial solution to conduct a more efficient neighborhood search. Besides, a graph-based neighborhood search scheme based on the dominated and non-dominated nodes is employed to search for a new candidate solution and to evaluate if it is a better solution. The proposed algorithms are important for decision-makers to utilize closed spaces more efficiently during the pandemic and minimize the risk of transmission of the pandemic.

This paper consists of the following sections. Section Literature Review presents a review of the literature on mathematical models and solution approaches for solving MDP problems, the simulated annealing algorithms applied to combinatorial problems, and the seat assignment model applied in various sectors. The formal problem formulation section presents the mathematical formulation of the MDPs model. Moreover, we discuss the framework details of the SA-MDPs developed for solving the problem. The computational experiments section presents the test runs of the SA-MDPs and discussions on their implementation. Finally, a summary of the problem and future research directions are presented.

## Literature review

The maximum diversity problem is known as the problem of choosing n items from a given m set to maximize the total distance or diversity among the chosen items. This problem is classified as NP-hard due to its combinatorial characteristic. Many real-life problems are modeled as MDP. Besides, due to the difficulty of solving MDP in an efficient computation time frame, many modeling techniques and heuristic and meta-heuristic algorithms have been presented. In this section, studies on MDP modeling and solution techniques are examined. In addition, studies on the Simulated annealing algorithm used for MDPs solution, which is a new application area of MDPs, have been reviewed. Eventually, seat assignment problems, which we classify as MDPs, were also examined.

MDP has been examined by many studies in the literature in terms of both modeling and solution methods. Kuby [10] proposed a mathematical model of the maximum diversity problem for locating *n* facilities among *m* candidate nodes to maximize total distance among the assigned locations. Dhir et al. [11] proposed a variety of integer programming models and counterpart models for the maximum diversity problem that can be applied to a range of fields. Palubeckis [12] presented an iterated tabu search algorithm to deal with the maximum diversity problem. Duarte and Marti [13] presented a tabu search algorithm with two constructive methods for the maximum diversity problem. The authors also integrated an improved local search and a short-term memory search procedure. Gallego et al. [14] developed a hybrid approach in the context of a scatter search algorithm to solve the maximum diversity problem. Porumbel et al. [15] presented a tabu search algorithm including local search based on a drop and add move procedure for the MDP. Marti et al. [16] examined a comprehensive study of comparing solution method, i.e. heuristic and metaheuristics to solve MDP. Wang et al. [17] proposed a tabu search algorithm to solve the maximum diversity problem in a reasonable amount of time. Parreno et al. [18] analyzed the four mathematical models, i.e. MaxMin, MaxSum, MaxMinSum, and MinDiff, for MDP to with the objective of maximizing diversity or distances. They recommended the use of the MaxSum model for MDP modeling in terms of computational efficiency and diversity.

Simulated annealing is a stochastic metaheuristic algorithm. SA has been successfully applied to many problems that have characteristics of combinatorial optimization by Delahaye et al. [19]. Mostly these studies have proposed specific local search and initial solution procedures due to the distinctive structure of each problem. Leite at al. [20] utilized the SA framework for a timetabling problem with an initial solution produced by the saturation degree heuristic. Wei et al. [21] applied SA with an open space-based algorithm for capacitated vehicle routing problems and loading. McKendal et al. [22] presented SA for solving the dynamic space allocation problem for allocating activities to workspaces and resources. Miao and Tian [23] employed a simulated annealing algorithm with an initial path selection algorithm for robot path planning in uncertain environments. Anand et al. [24] presented a Simulated annealing algorithm-based heuristic to minimize the unused area in the floor planning with the objective of the circuit design process.

The problem of seat assignment or allocation, considering social distancing in pandemics, has been extensively studied in various sectors like transportation, health, and education. Murray [25] developed a spatial optimization model to assign students to classrooms with consideration of social distancing. Karakose and Dundar [26] proposed four mathematical models along with an efficient frontier algorithm to maximize total distance while assigning students to seats in classrooms. However, they depended on the commercial solver to solve the problem. Haque and Hamid [27] presented a mixed integer programming model to maximize revenue in the rail seat assignment problem while keeping the social distance among the passengers. Xu et al. [28] introduced a seat allocation model to maximize revenue in rail transportation by considering a constraint of social distance. Bortelete et al. [29] developed a decision support platform to allocate students to classroom seats. More precisely, they proposed two mathematical models for fixed seats to maximize the assigned students and the minimum distance between the occupied seats. Moore et al. [30] developed a mixed integer programming model of seat assignment problem for transit vehicles(i.e., buses, airplanes, and trains) while considering physical distance among the passengers. The authors, specifically, took into account household grouping in seat assignment to increase the utilization rate of vehicle’ capacity. Murray and Burtner [31] proposed GIS and spatial-based location models to optimize office space occupancy and room seating while considering physical distancing to reduce disease transmission. Lokhande et al. [32] developed a GIS-based decision support system to seat students in classrooms. Fischetti et al. [33] investigated the best placement of seats or tables in a common area to reduce the transmission of viruses. The authors strive to place seats or tables at the optimum location, taking into account the social distance constraint, with a modeling approach similar to the problem of placing offshore wind turbines. Contardo and Costa [34] proposed a set packing problem that would maximize the seating arrangement in the dining room, by considering the social distance rule, customers’ seating wishes, the capacity of the tables, and the positions of the chairs. As far as we know, this study is the first to examine the seat assignment model with social distancing constraint in the context of MDP with a simulated annealing algorithm approach to solve in a reasonable CPU time.

## Formal problem formulation

The Maximum Diversity Problem(MDP) originally consisted of a quadratic objective function. In MDP, a predetermined n number of instances are chosen from m number of sets(V), n<m, which are located to certain places to maximize distance among them. In formulation, let V be set of items, and i,j∈V, *d*_*ij*_ is the distance between instance i and j. Here, *x*_*i*_ is a binary variable if the instance i is selected *x*_*i*_ is taking the value of 1, otherwise 0. The objective function 1 consists of quadratic term *x*_*i*_*x*_*j*_. The constraint 2 forces to assign n number of elements.

$$\begin{array}{c} \text{max}\;\sum_{(i,j)\in V|j>i}d_{ij}x_{i}x_{j} \end{array}$$ (1)

$$\begin{array}{c} \text{subject}\;\text{to}\;\sum_{i\in V}x_{i}=n \end{array}$$ (2)

$$\begin{array}{cc} x_{i},x_{j}\in \left\{0,1\right\}\; & \forall i,j\in V \end{array}$$ (3)

The quadratic function in the objective 1 can be reformulated with a newly introduced variable, *ω*_*ij*_, where if seat *i* and *j* assigned, it takes the value of 1, otherwise 0. The reformulation of MDP into the mixed integer programming introduced by [35]. This has facilitated the solving of MDP.

Below we introduce MPDs model, in which n number of seats are selected among |*V*| capacity of the classroom in order to assign students to these n seats in an optimum way while considering the social distance among the students. Assigning students to seats in an optimum way while keeping the social distance among the students could reduce the infection risk. In MDPs, *z*_*i*_ and *z*_*j*_ are binary variables and if a student is assigned to seat *i* or *j*, taking a value of 1, otherwise 0. The distance between each seat *i* and *j* are demonstrated by *ϱ*_*ij*_, where due to symmetry in the distance matrix, *i* < *j*. Let *η* be the social distance that needs to be kept while assigning students to seats. The mathematical model defined below maximizes the total distance among the students and disperses them as far as away while keeping the physical distance between the students.

$$\begin{array}{cc} \text{max}\; & \sum_{(i,j)\in V|j>i}\varrho_{ij}\omega_{ij} \end{array}$$ (4a)

$$\begin{array}{ccc} \text{s.t.}\; & \omega_{ij}\leq z_{i},\; & \forall i,j\in V|j>i \end{array}$$ (4b)

$$\begin{array}{ccc} & \omega_{ij}\leq z_{j}, & \forall i,j\in V|j>i, \end{array}$$ (4c)

$$\begin{array}{ccc} & z_{i}+z_{j}\leq \omega_{ij}+1, & \forall i,j\in V|j>i \end{array}$$ (4d)

$$\begin{array}{ccc} & \omega_{ij}\leq \frac{\varrho_{ij}}{\eta }, & \forall i,j\in V|j>i \end{array}$$ (4e)

$$\begin{array}{cc} & \sum_{i\in V}z_{i}=\vartheta \end{array}$$ (4f)

$$\begin{array}{ccc} & z_{i},z_{j},\omega_{ij}\in \left\{0,1\right\}\; & \forall i,j\in V \end{array}$$ (4g)

The objective (4a) of MDPs model maximizes the overall distance among the assigned seats. When both seat *i* and seat *j* are filled, the constraints (4b)–(4d) enforces *ω*_*ij*_ variable to have the value of 1. If the distance between seats *i* and *j* is not higher than or equal to *η*, constraint (4e) ensures that neither seats *i* nor *j* can be assigned simultaneously. The constraint (4f) allocates *ϑ* number of students to the classroom.

We can solve the above formulation and get a feasible solution. However, these formulations are valid for the small number of |V|. When the number of *m* and *n* increases, the problem becomes intractable to solve. Hence, a heuristic or metaheuristic algorithm would be needed to provide an optimal or near-optimal solution in a reasonable computational time.

## The simulated annealing framework approach

The MDPs is classified as NP-Hard problem. Therefore, while the number of instances of seats increases, the problem can be intractable in terms of reasonable computational time and obtaining a quality solution. Despite being simple to formulate, this problem is challenging to determine the optimal solution because of the inherent combinatorics. The number of solutions to take into account for n students to assign m capacity classroom is $\left(\begin{array}{c} m\\ n \end{array}\right)$, and this number increases extremely quickly as n and m increases. The overall architecture of the Simulated annealing algorithm is provided in Algorithm 1. The SA starts with a feasible solution $\ddot{\text{z}}$, details are provided in Algorithm 3 in the subsection. Note also that to ascertain the parameter settings of SA, both theoretical and experimental methods are utilized. The details of the input parameters of SA are as follows. T is the temperature value and acts as the outer loop in SA. *c* is the cooling ratio that reduces the temperature by a constant rate, which is 0 < *c* < 1. *k* is a constant value used in computing probability to accept the worse solution or not. *η* is the social distance predetermined by decision-makers. *ϑ* is the number of students to be assigned to the classrooms. Note that *ϑ* should be less than or equal to the maximum number of students that can be fitted in the classrooms, which is detailed in the study of Dundar and Karakose [8]. *ξ* is the number of inner loop iterations for each temperature T. In line 4 the new solution $\ddot{\text{z}}$ is defined with respect to a function of the neighborhood search procedure as the steps demonstrated in Algorithm 4. In line 5, $\ddot{\omega }$ is updated based on UpdateFunction with $\ddot{\text{z}}$, the details of steps are provided in Algorithm 2. Between line 8 and 16 the metropolis criterion has been applied to accept some local optimal solutions that are worse than the current best solution $\tilde{\text{z}}$ with random probability *r* in case of $\ddot{F}<\tilde{F}$, namely Δ < 0, where $\ddot{F}$ is the new objective function and $\tilde{F}$ is incumbent objective function value. The probability of accepting the worse solution is estimated based Equation.

$$\begin{array}{c} P=min(e^{\frac{\Delta }{k*T}},1) \end{array}$$ (5)

If the neighborhood solution $\ddot{\text{z}}$, is better that the best solution z*, the best and current solution $\left(\tilde{z}\right)$ are updated.

**Algorithm 1** Simulated Annealing algorithm framework

1: **Inputs**:

T,k,c,η,ϑ,ξ

$\ddot{z}\leftarrow InitialSolution\left(seat-cord,\eta ,\vartheta \right)$

2: **while** ϵ≤T **do**

3:  **for** *i* ∈ *range*(*ξ*) **do**

4:   $\ddot{z}\leftarrow NeighboorhodSearch\left(\tilde{z}\right)$

5:   $\ddot{\omega }\leftarrow UpdateFunction\left(\ddot{z}\right)$

6:   $\ddot{F}\leftarrow ObjFunction\left(\ddot{\omega }\right)$

7:   $\Delta \leftarrow \ddot{F}-\tilde{F}$

8:   **if** $\ddot{F}<\tilde{F}$ **then**

9:    *r* ← *random*(0, 1)

10:    $P\leftarrow min(e^{\frac{\Delta }{k*T}},1)$

11:    **if** P≥r **then**

12:     *Accept* ← *True*

13:    **else**

14:     *Accept* ← *False*

15:    **end if**

16:   **else**

17:    *Accept* ← *True*

18:   **end if**

19:   **if** *Accept* = *True* **then**

20:    $\tilde{z}\leftarrow \ddot{z}$

21:    $\tilde{F}\leftarrow \ddot{F}$

22:   **end if**

23:   **if** $\ddot{F}>F^{*}$ **then**

24:    $z^{*}\leftarrow \ddot{z}$

25:   **end if**

26:  **end for**

27:  T=T*c

28: **end while**

29: **return**: z*

**Algorithm 2** UpdateFunction procedure

1: **for** *i* ∈ *V* **do**

2:  **for** *j* ∈ *V* **do**

3:   **if** ${\ddot{z}}_{i}=1$ and ${\ddot{z}}_{j}=1$ and *i* < *j* **then**

4:    *ω*_*ij*_ = 1

5:   **else**

6:    *ω*_*ij*_ = 0

7:   **end if**

8:  **end for**

9: **end for**

### Evaluation function

The objective function can be modified by adding a term known as the Lagrange multiplier, λ ≥ 0, to the constraint including social distance. ObjFunction F as described in the below equation is augmented by the constraint that is multiplied by nonnegative.

$$F(\omega ,\lambda )=\sum_{i,j\in I|j>i}\varrho_{ij}\omega_{ij}-\text{max}\left(0,\lambda_{ij}*\sum_{i,j\in I|j>i}\omega_{ij}-\varrho_{ij}\eta \right)$$ (6)

### Feasible initial solution

A good initial solution enhances the efficiency of the SA algorithm in finding optimal or near-optimal solutions. Hence, we create a quality initial solution based on the greedy approach by fitting the pre-determined number of students in a classroom while maintaining social distance. In the first step, the algorithm determines the coordinate point of each seat and randomly selects a seat in the classroom. Depending on the first seat assigned, the greedy algorithm iteratively chooses the seat that maximizes the total distance.

**Algorithm 3** Greedy-random based initialization algorithm

1: **Inputs**:

ϑ,η,M

2: $D_{n}=\left\{\right\}$, ${\bar{D}}_{n}=\left\{\right\}$, K=random(V),N=G.nodes()

3: **for** i∈K **do**

4:  $D_{n}=D_{n}\cup i$

5:  ${\bar{D}}_{n}|=G.neighbors\left(i\right)$

6: **end for**

7: $S_{c}=N-D_{n}-{\bar{D}}_{n}$

8: |ϑ|=|ϑ|-|K|

9: **while** *ϑ* ≠ 0 and $S_{c}\neq 0$ **do**

10:  *t*_*best*_ = 0

11:  **for** $i\in S_{c}$ **do**

12:   *t* = 0

13:   **for** $j\in D_{n}$ **do**

14:    $t=t+M_{ij}$

15:   **end for**

16:   **if** *t* > *t*_*best*_ **then**

17:    *t*_*best*_ = *t*

18:    i* = *i*

19:   **end if**

20:  **end for**

21:  $S_{c}=S_{c}\backslash i^{*}$

22:  ${\bar{D}}_{n}^{*}=G.neighbors\left(i^{*}\right)-D_{n},$

23:  $D_{n}=D_{n}\cup i^{*}$

24:  $D_{n}=D_{n}\cup {\bar{D}}_{n}^{*}$

25:  $S_{c}=S_{c}-{\bar{D}}_{n}^{*}$

26:  |*ϑ*| = |*ϑ*| − 1

27: **end while**

28: **return**: feasible initial solution

In Algorithm 3, details of steps followed to generate a feasible initial solution for SA-MDPs are provided. In lines 1–2, input parameters are presented, which are the sets of seats, V, social distance *η*, the number of students will be assigned, *ϑ*, distance matrix of seats, M. N is the set of all seats determined based on the network nodes procedure. A randomly chosen seat, K, is initially located then accordingly the rest of the students are assigned based on a greedy approach of maximizing the distance between each student pair. In lines 3–6, depending on the set K, Dominating seats, $D_{n}$, Dominated seats, ${\bar{D}}_{n}$, are determined. In line 7 candidate seats, $S_{c}$ are identified by subtracting the dominating and dominated notes from all seat sets N. In line 8, the number of assigned students *ϑ* is updated by subtracting the assigned seats set K. In line 9–20, while *ϑ* number of students are assigned to seats from $S_{c}$ are not equal to zero, the loop operations continue to assign the students to seats. In each iteration, the seats that maximize total distance are chosen. Between lines 21–26 and based on the chosen seat, $S_{c}$, $D_{n}$, and ${\bar{D}}_{n}$ are updated.

### Neighborhood search

To perform a feasible search, the seats are divided into dominating seats, $D_{n}$, dominated seats, ${\bar{D}}_{n}$, and candidate seats for assignment,$S_{c}$, by using the graph theory concept. Based on the incumbent solution, the assignable seats and non-assignable seats are determined. A swap operation is conducted between the assigned and candidate seat sets. A seat is randomly selected among the assigned seats and its value is changed from 0 to 1. Similarly, a random seat is picked among the assigned seats, its value is changed from 1 to 0. In algorithm 4 details are provided. In the algorithm, N is the set of all notes, i.e set of seats, E is the set of edges between seats if the distance between seats is less than the predetermined social distance. *e*_*ij*_ is used to show that there is a link between seat *i* and *j*. In algorithm 4, in the line between 2–8, the sets of edges are determined based on the social distance. In other words, if the seats are in the proximity of the social distance of an assigned seat, an edge is created and appended to the sets of E. In line 9, the sets of all possible edges are created by utilizing graph theory concept. Between line 10–14, if the incumbent solution, ${\tilde{z}}_{j}$ equals 1, i.e. already assigned, the seats are categorized into the assigned seat list, $S_{a}$. In line 15, a seat is randomly chosen among $S_{a}$, and its value is changed from 1 to 0. This seat is popped from the assigned list $S_{a}$. In line 17–20, the dominating set $D_{n}$ and ${\bar{D}}_{n}$ is determined by considering the assigned seat list $S_{a}$. As described in the line 21, the dominated nodes $D_{n}$ and dominating nodes ${\bar{D}}_{n}$ are extracted from all seats N, to determine the candidate seats $S_{c}$ for the assignment. In line 22, a seat is randomly chosen among the $S_{c}$ and assigned a value of 1. Finally the the new candidate solution, $\ddot{\text{z}}$ is returned.

**Algorithm 4** Graph-based neighborhood search procedure

1: **Inputs**:

$\tilde{z}$ , $E=\left\{\right\},S_{a}=\left\{\right\},S_{c}=\left\{\right\}$

2: **for** i∈V **do**

3:  **for** j∈V **do**

4:   **if** *i* < *j* and $\varrho_{ij}<\eta$ **then**

5:    $E=E\cup e_{ij}$

6:   **end if**

7:  **end for**

8: **end for**

9: G.addedgesfrom(E)

10: **for** j∈V **do**

11:  **if** ${\tilde{z}}_{j}==1$ **then**

12:   $S_{a}=S_{a}\cup {\tilde{z}}_{j}$

13:  **end if**

14: **end for**

15: $random(S_{a})=0$

16: $D_{n}=\left\{\right\}$, ${\bar{D}}_{n}=\left\{\right\},N=G.nodes\left(\right)$

17: **for** $i\in S_{a}$ **do**

18:  $D_{n}=D_{n}\cup i$

19:  ${\bar{D}}_{n}|=G.neighbors\left(i\right)$

20: **end for**

21: $S_{c}=N-D_{n}-{\bar{D}}_{n}$

22: $random(S_{c})=1$

23: **return**: $\ddot{z}$

**Fig 1:**
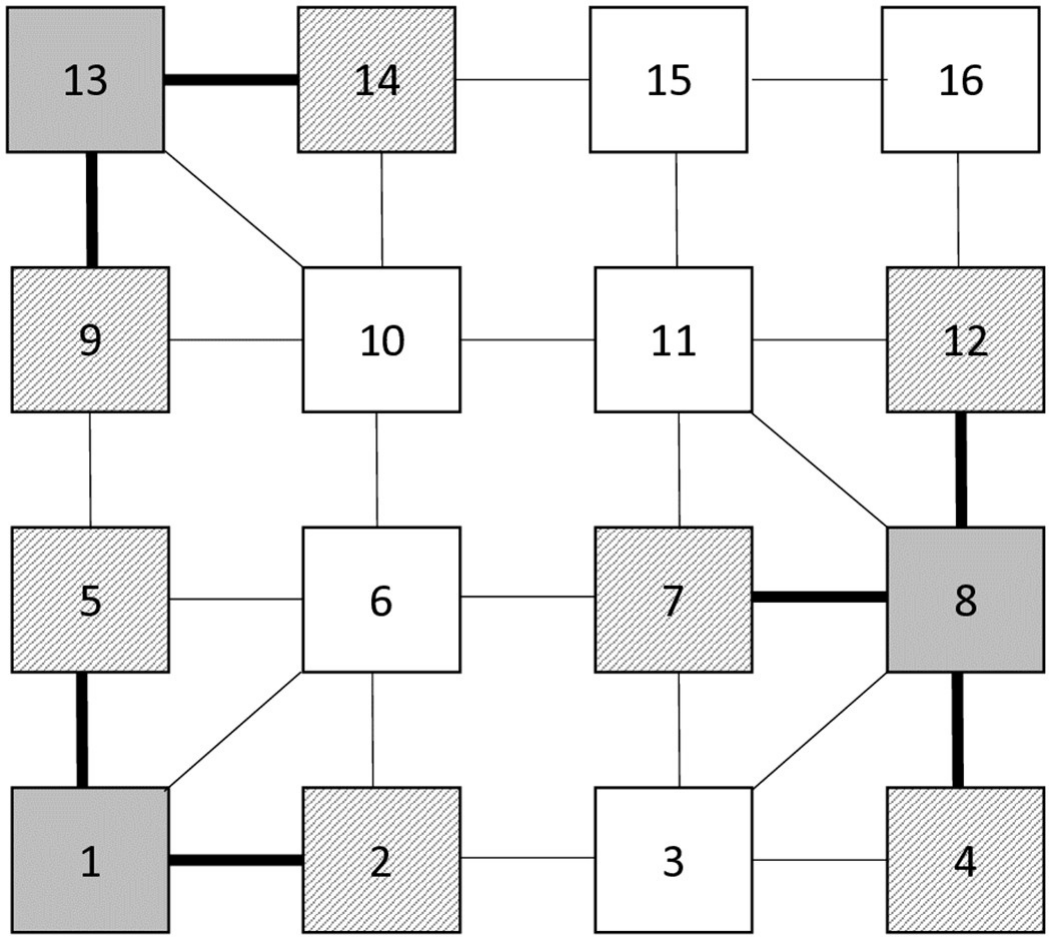
An example to illustrate the neighborhood search procedure.

For example, as provided in Fig 1, the exemplified classroom has 16 seats. Let’s assume that the distance between seats on the same x or y axes is equal and 90 cm, while the distance between seats connected on a diagonal is about 127 cm. In this example, the dominating seats $D_{n}$ are the seats with numbers 1, 8, and 13, and the dominated nodes ${\bar{D}}_{n}$ are connected to dominating nodes, which are in proximity of them. The dominated nodes are determined based on pre-defined social distances of 100 cm, which are 2, 4, 5, 7, 12, 9, and 14. On the other hand, the undominated nodes, i.e. unassigned seats, are 3, 6, 10, 11, 15, and 16.

## Computational experiments

The classrooms with different sizes of capacities are considered to test the performance of the Simulated Annealing algorithm for MDPs (SA-MDPs). The algorithms were coded in Python and tested with a computer Intel i5–7500 CPU 3.40 GHz processor and 8 GB RAM. We used data gathered from a spectrum of classrooms at Bartin University. The data from the classrooms with different capacities are considered, including the smallest capacity classroom and the highest capacity classroom. The smallest classroom has a capacity of 30 seats while the largest classroom has a capacity of 160 seats. When calculating the distance between two seats the Euclidean distance method was utilized by taking into account the center point of the seats. The maximum number of students that can be assigned under the required social distancing for each classroom was determined by considering the study of Dundar and Karakose (2023). That study also identified the social distance scenarios so that the model could return a feasible solution.

In Table 1, the range of parameters utilized for setting the optimal computational environment of SA-MDPs is given. We run SA-MDPs based on each scenario of the combination of T, *ξ*, and *c*. The minimum value of T=50, with an increased rate of 50 for each fixed value of *ξ*, and *c*, considered to test the effect of T on the best results and the CPU time. SA-MDPs is tested for *ξ* range from 100 to 1000 with an increase of 100 for each fixed value of T, and *c*. Moreover, *c* value with a range of 0.9 and 0.99 with an increment rate of 0.01 simulated how the cooling process of T impacts the optimal solution and CPU time. After many preliminary test calculations, we finally determined T = 50, *c* = 0.95, and *ξ* = 100 as SA-MDPs parameters. Each scenario of SA-MDPs is simulated 100 times to observe how the solution deviates. We determine the best value, the worst value, and the average CPU time based on 100 simulations.

**Table 1:** The range used to determine optimal SA-MDPs parameters for computational testings.

	Min Value	Max Value	Increment rate
Social Distance (*η*)	100	200	50
T	50	500	50
*ξ*	100	1000	100
c	0.9	0.99	0.01

To test the proposed SA-MDPs in terms of solution and computational time and to compare it with CPLEX, we considered the classroom-coded EO-K2–5, which has the lowest capacity with 30 seats among the classrooms. As given in Table 2, SA-MDPs were tested with a combination of *ϑ* = 3, 6, and 9 students and social distances (*η*) starting from 100 cm with an increment of 50 cm. In the given table, the best and worst solution returned by the SA-MDPs, and their average CPU time are provided for 100 simulations based on the aforementioned SA-MDPs input parameters. The best solution found by SA-MDPs for each scenario is the same as the optimal value found by CPLEX. Note that there is no deviation for the best and worst solution provided by SA-MDPs for the scenario of *ϑ* = 3 and *η* = 100, 150, and 200 cm. When the number of students is increased to 6 and 9, a deviation is observed between the best and the worst solution due to the increase in social distance. In this small-capacity classroom, SA-MDPs generally returns the optimum solution in a shorter time than CPLEX under the specified parameters. Especially in terms of computational time when the number of students and social distance increases, SA-MDPs provides a solution approximately 10 times faster than CPLEX.

**Table 2:** Comparing the optimal values and CPU time for a classroom coded with EO-K2–5.

SA-MDPs					CPLEX	
# of students *ϑ*	Social Distance (*η*)	Best Value (cm)	Worst Value (cm)	Average CPU Time (sec)	Optimal value(cm)	CPU Time (sec)
3	100	1509	1509	1.7	1509	5
	150	1509	1509	2.0	1509	4
	200	1509	1509	2.1	1509	4
6	100	6393	6383	1.9	6393	26
	150	6301	6118	2.1	6301	5
	200	6301	4431	2.1	6301	2
9	100	13944	13944	1.9	13944	23
	150	12947	10833	2.2	12947	2
	200	-	-	-	-	-

The classroom coded with EA-K1–5 has a capacity of 69 seats. We consider it a mid-size classroom to test SA-MDPs. The computational results of SA-MDPS and CPLEX are given in Table 3. As observed from the table, for *ϑ* = 3, and *η* = 100, 150, and 200, SA-MDPs and CPLEX produce the same optimal solution. However, SA-MDPs finds the optimal solution almost 15 times faster than CPLEX. Moreover, for *ϑ* = 6, and *η* = 100 and 150, SA-MDPs and CPLEX provide the same solution, but in terms of CPU time, CPLEX returns that solution in a predetermined time limit of 3600 sec. In the scenario of *ϑ* = 6, and *η* = 200, *ϑ* = 9, and *η* = 200, *ϑ* = 12, and *η* = 200 both SA-MDPs and CPLEX find proven optimal solutions. In these cases, however, CPLEX provides the optimal solution in a time of less than one-hour limit. The reason for finding an optimal solution in a short time in these scenarios is that, as stated in the graph-based neighborhood search procedure, fewer nodes are taken into account for the assignment scenario, as the search space decreases considerably. In the scenario of *ϑ* = 9 and *η* = 100, 150 cm, SA-MDPs gives the same best solution that CPLEX returns. Nevertheless, in these scenarios, SA-MDPs provides the best solution in a shorter time. For *ϑ* = 12 and *η* = 100, 150 cm, SA-MDPs returns a better best solution than CPLEX.

**Table 3:** Comparing the optimal values and CPU time for a classroom coded with EA-K1–5.

SA-MDPs					CPLEX	
# of students *ϑ*	Social Distance (*η*)	Best Value (cm)	Worst Value (cm)	Average CPU Time (sec)	Optimal value(cm)	CPU Time (sec)
3	100	2209	2209	11	2209	165
	150	2209	2209	12	2209	113
	200	2209	2209	13	2209	123
6	100	9535	9535	12	9535	>3600
	150	9259	9146	12	9259	>3600
	200	9259	9253	13	9259	2752
9	100	21011	21011	12	20897	>3600
	150	19816	19666	12	19807	>3600
	200	19807	19807	13	19807	662
12	100	37086	37086	12	36506	>3600
	150	33855	33625	12	33679	>3600
	200	31996	27966	13	31996	479

SA-MPDs is tested for EA-Z-10 coded classroom with a capacity of 116 seats, and the computational results are shown in Table 4. Pre-experimental runs in CPLEX showed us that it would take hours to find an optimum solution and in most cases RAM was insufficient. Therefore, we applied a one-hour time limit for CPLEX to produce the best solution it finds. We noticed that for almost each combination scenario of *ϑ* and *η*, SA-MDPs provides a better solution. As can be seen from Table 4, based on the applied time limit, SA-MPDs finds a better solution up to %4. For instance, for *ϑ* = 5 and *η* = 200 cm, SA-MPDs finds the best value of 9096, while CPLEX finds 8877 cm. Besides this, in terms of CPU time, SA-MDPs finds the solution with an average CPU time of 34 sec, but on the other hand, it took a one-hour CPU time to find that solution for CPLEX.

**Table 4:** Comparing the optimal values and CPU time for a classroom coded with EA-Z-10.

SA-MDPs					CPLEX	
# of students *ϑ*	Social Distance (*η*)	Best Value (cm)	Worst Value (cm)	Average CPU Time (sec)	Optimal value(cm)	CPU Time (sec)
5	100	9307	9307	31	9245	>3600
	150	9294	9105	34	9203	>3600
	200	9096	9096	34	8877	>3600
10	100	36741	36408	31	35614	>3600
	150	35889	35523	36	35578	>3600
	200	34232	34213	33	34321	>3600
15	100	80025	79382	31	76975	>3600
	150	76959	75708	32	76236	>3600
	200	68825	61884	34	69046	2201

**Fig 2:**
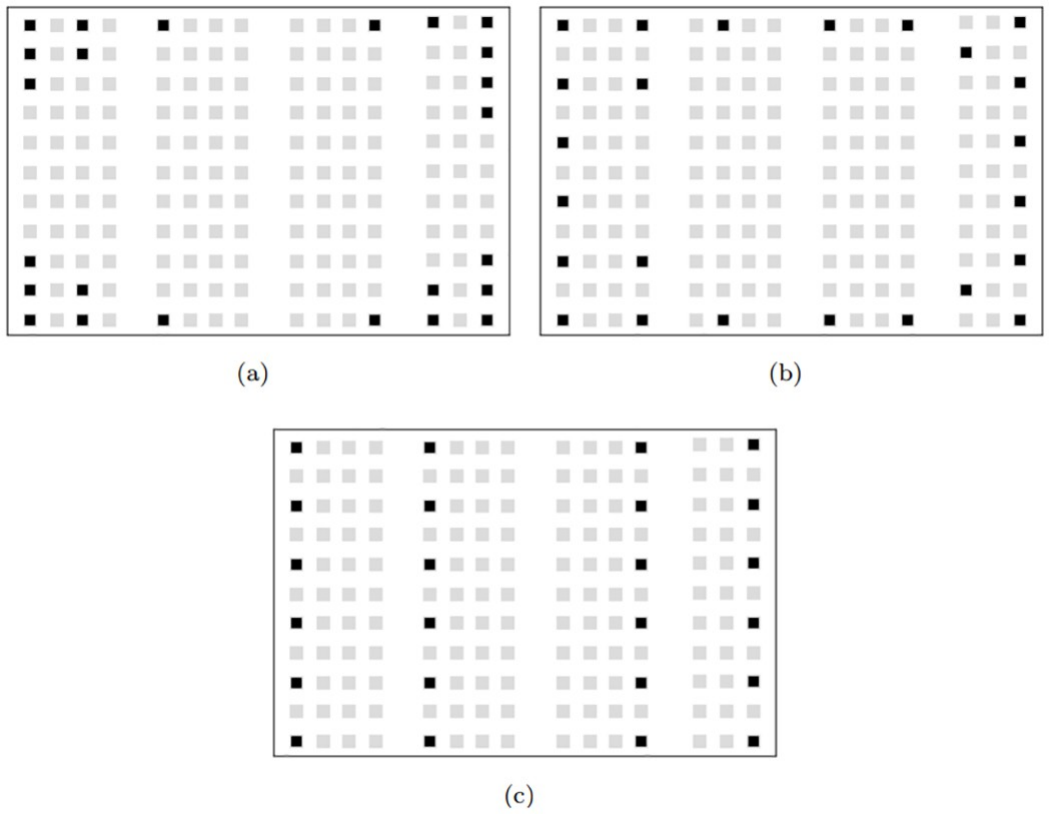
The best optimal layout found by SA-MDPs for a ED-K1–11/B classroom with 165 seats capacity (a) *ϑ* = 24 and *η* = 100 cm assignment scenario (b) *ϑ* = 24 and *η* = 150 cm assignment scenario (c) *ϑ* = 24 and *η* = 200 cm assignment scenario.

The classroom coded ED-K1–11/B is the largest one considered for the computational testing with a capacity of 164 seats. For the computational testing of this classroom, the number of students to assign classroom changes in the range of 6, and 24 with an increment rate of 6 for the social distance from 100 cm to 200 cm with an increment of 50 cm. As given in Table 5 for *ϑ* = 6, and social distance of 100 cm the SA-MDPs provides a maximum distance of 14738 cm with an average computational time of 62 sec. Notice that for each combination of the number of students and the social distance SA-MDPs find a better solution within the time limit of one hour of computational time. Moreover, another observation is that for each student scenario to be assigned, the best value found decreased when the social distance increased. The reason for this decrease in the objective function value is that as social distance increases, students at farther distances begin to be assigned closer to each other. In the *ϑ* = 6 and *η* = 200 assignment scenario, the best solution found by SA-MDPs is about %2 better than the CPLEX returned within the one-hour time limit. When *ϑ* increases from 6 to 9 for *η* = 100 cm, the best value found by SA-MDPs is %12 is higher than the CPLEX returned value within the time limit. As seen in Table 5, while the difference between the worst and the best value found by SA-MDPs is 8 for *ϑ* = 18 and *η* = 200 assignment scenario, this difference increases to 12467 cm for *ϑ* = 24 and *η* = 200 assignment scenario. The best seating layout for *ϑ* = 24 and *η* = 100, 150, and 200 cm found by SA-MDPs is demonstrated in Fig 2. The reason for the differences in seating arrangements in Fig 2 is that the number of students to be assigned is lower than optimal capacity and social distances. While the social distance in sub-figure (a) and (b) is 100 cm and 150 cm, respectively, it is 200 cm in the sub-figure (c). As mentioned before, the social distance may differ from country to country depending on the decision made by the institutions. These cases were originally added to demonstrate an example of the seating arrangement that can occur in the classrooms when the number of students to be assigned and social distance restrictions vary. As the number of students to be assigned is *ϑ* = 24 and the social distance is *η* = 100 cm, the total distance as the objective function is 193,803 cm. While we make the social distance 150 cm, the total distance becomes 202,876 cm. On the other hand, when the social distance is 200 cm, the total distance decreases to 184,105 cm. In this case, since keeping the social distance 150 cm maximizes the total distance, it can be the most ideal seating arrangement in which the risk of general virus transmission is lowest.

**Table 5:** Comparing the optimal values and CPU time for a classroom coded with ED-K1–11/B.

SA_MDPs					CPLEX	
# of students *ϑ*	Social Distance (*η*)	Best Value (cm)	Worst Value (cm)	Average CPU Time (sec)	Optimal value(cm)	CPU Time (sec)
6	100	14738	14705	62	14508	>3600
	150	14551	14395	63	13605	>3600
	200	14481	14424	65	13943	>3600
12	100	58387	57894	61	52348	>3600
	150	56161	55569	63	54931	>3600
	200	55605	55346	65	53953	>3600
18	100	127140	126369	61	112514	>3600
	150	121185	120089	64	116066	>3600
	200	116326	116318	65	116318	>3600
24	100	220443	219998	62	193803	>3600
	150	207612	204061	64	202876	>3600
	200	187699	175232	66	184105	>3600

**Fig 3:**
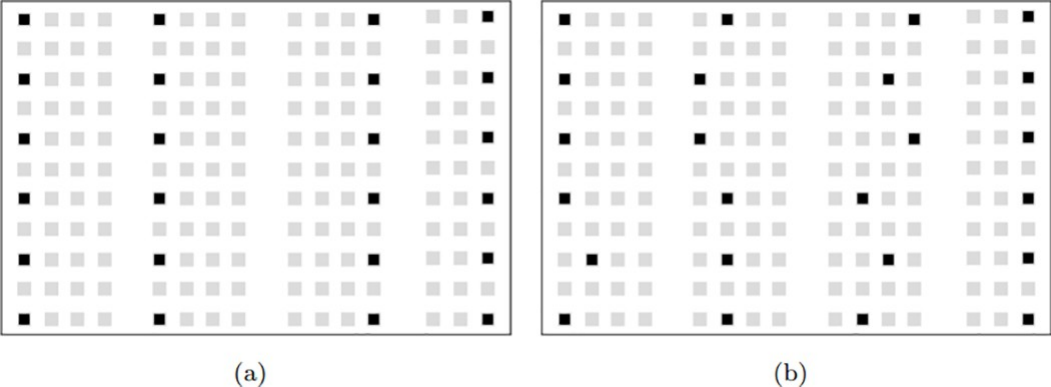
Comparing the best optimal layout found by SA-MDPs and CPLEX for a ED-K1–11/B classroom (a) The best optimal layout found by SA-MDPs (b) The best optimal layout found by CPLEX.

In Fig 3, we compare the best solution provided by SA-MDPs with the solution found by CPLEX for the assignment scenario of *ϑ* = 24 and *η* = 200 cm. As aforementioned, we applied the limit of one hour for CPLEX to return a solution. The best optimal objective value found by CPLEX is 184105 cm, while SA-MDPs provided the objective value of 187699.

## Conclusion

In this study, a novel application of the maximum diversity problem has been proposed to assign students to classroom seats to maximize total distance while keeping predetermined physical distancing among the students. We name the new model Maximum diversity social distancing problem (MDPs). MDPs is considered as NP-hard problems, so we proposed a Simulated Annealing Algorithm framework for MDPs(SA-MDPs) to determine optimal or near-optimal solutions within a reasonable computational time. In the proposed SA-MDPs, to start with a feasible solution we presented the greeedy-random algorithm. For the neighborhood searching, the graph-based neighborhood search procedure has been introduced. The proposed SA-MDPS was tested with data from classrooms of different capacities. The computational test showed that the proven optimal results are obtained in a short time for the small capacity classes. In larger capacity classrooms, within the one-hour time constraint, SA-MDPs provided the best results, better than CPLEX, in almost all scenarios.

## Supporting information

S1 Data Supporting information file containing additional data used in the study. (DOCX)
