# Supplementary material for: A simulated annealing with graph-based search for the social-distancing problem in enclosed areas during pandemics
Source: PLoS One. 2025 Feb 11;20(2):e0318380. doi: 10.1371/journal.pone.0318380 (PMC11813157; doi:10.1371/journal.pone.0318380)
Supplement: S1 Data — (DOCX) [file pone.0318380.s001.docx]

The following data include the codes of the classrooms, their capacities, and the x-y coordinates of the seats. In this article, the data from the classrooms EO-K2-5, EA-K1-5, EA-Z-10, and ED-K1-11/B were used.

**Classroom Code:** EO-K2-5

**Classroom capacity:** 30 seats

x-y coordinates of seats:

{0:[50,50],1:[108,50],2:[337,50],3:[390.75,50],4:[444.5,50],5:[498.25,50],6:[337,156.875],7:[390.75,156.875],8:[444.5,156.875],9:[498.25,156.875],10:[50,159],11:[108,159],12:[337,263.75],13:[390.75,263.75],14:[444.5,263.75],15:[498.25,263.75],16:[50,268],17:[108,268],18:[337,370.625],19:[390.75,370.625],20:[444.5,370.625],21:[498.25,370.625],22:[50,377],23:[108,377],24:[337,477.5],25:[390.75,477.5],26:[444.5,477.5],27:[498.25,477.5],28:[50,486],29:[108,486]}

**Classroom Code:** EA-K1-5

**Classroom capacity:** 69 seats

x-y coordinates of seats:

{0:[50,50],1:[105,50],2:[160,50],3:[268,50],4:[323,50],5:[378,50],6:[494,50],7:[549,50],8:[604,50],9:[50,155],10:[105,155],11:[160,155],12:[268,155],13:[323,155],14:[378,155],15:[494,155],16:[549,155],17:[604,155],18:[50,260],19:[105,260],20:[160,260],21:[268,260],22:[323,260],23:[378,260],24:[494,260],25:[549,260],26:[604,260],27:[50,365],28:[105,365],29:[160,365],30:[268,365],31:[323,365],32:[378,365],33:[494,365],34:[549,365],35:[604,365],36:[50,470],37:[105,470],38:[160,470],39:[268,470],40:[323,470],41:[378,470],42:[494,470],43:[549,470],44:[604,470],45:[50,575],46:[105,575],47:[160,575],48:[268,575],49:[323,575],50:[378,575],51:[494,575],52:[549,575],53:[604,575],54:[50,680],55:[105,680],56:[160,680],57:[268,680],58:[323,680],59:[378,680],60:[494,680],61:[549,680],62:[604,680],63:[50,785],64:[105,785],65:[160,785],66:[494,785],67:[549,785],68:[604,785]}

**Classroom Code:** EA-Z-10

**Classroom capacity: 110 seats**

x-y coordinates of seats:

{0:[50,50],1:[103.5,50],2:[157,50],3:[210.5,50],4:[354,50],5:[407.5,50],6:[461,50],7:[514.5,50],8:[649,50],9:[702.5,50],10:[756,50],11:[809.5,50],12:[50,164.75],13:[103.5,164.75],14:[157,164.75],15:[210.5,164.75],16:[354,164.75],17:[407.5,164.75],18:[461,164.75],19:[514.5,164.75],20:[649,164.75],21:[702.5,164.75],22:[756,164.75],23:[809.5,164.75],24:[50,279.5],25:[103.5,279.5],26:[157,279.5],27:[210.5,279.5],28:[354,279.5],29:[407.5,279.5],30:[461,279.5],31:[514.5,279.5],32:[649,279.5],33:[702.5,279.5],34:[756,279.5],35:[809.5,279.5],36:[50,394.25],37:[103.5,394.25],38:[157,394.25],39:[210.5,394.25],40:[354,394.25],41:[407.5,394.25],42:[461,394.25],43:[514.5,394.25],44:[649,394.25],45:[702.5,394.25],46:[756,394.25],47:[809.5,394.25],48:[50,509],49:[103.5,509],50:[157,509],51:[210.5,509],52:[354,509],53:[407.5,509],54:[461,509],55:[514.5,509],56:[649,509],57:[702.5,509],58:[756,509],59:[809.5,509],60:[50,623.75],61:[103.5,623.75],62:[157,623.75],63:[210.5,623.75],64:[354,623.75],65:[407.5,623.75],66:[461,623.75],67:[514.5,623.75],68:[649,623.75],69:[702.5,623.75],70:[756,623.75],71:[809.5,623.75],72:[50,738.5],73:[103.5,738.5],74:[157,738.5],75:[210.5,738.5],76:[354,738.5],77:[407.5,738.5],78:[461,738.5],79:[514.5,738.5],80:[649,738.5],81:[702.5,738.5],82:[756,738.5],83:[809.5,738.5],84:[50,853.25],85:[103.5,853.25],86:[157,853.25],87:[210.5,853.25],88:[354,853.25],89:[407.5,853.25],90:[461,853.25],91:[514.5,853.25],92:[649,853.25],93:[702.5,853.25],94:[756,853.25],95:[809.5,853.25],96:[50,968],97:[103.5,968],98:[157,968],99:[210.5,968],100:[354,968],101:[407.5,968],102:[461,968],103:[514.5,968],104:[50,1082.75],105:[103.5,1082.75],106:[157,1082.75],107:[210.5,1082.75],108:[354,1082.75],109:[407.5,1082.75],110:[461,1082.75],111:[514.5,1082.75],112:[50,1197.5],113:[103.5,1197.5],114:[157,1197.5],115:[210.5,1197.5]}

**Classroom Code:** ED-K1-11/B

**Classroom capacity: 165 seats**

x-y coordinates of seats:

{0:[50,50],1:[104,50],2:[158,50],3:[212,50],4:[326,50],5:[380,50],6:[434,50],7:[488,50],8:[603,50],9:[657,50],10:[711,50],11:[765,50],12:[886,50],13:[941.3333333333334,50],14:[996.6666666666667,50],15:[50,154],16:[104,154],17:[158,154],18:[212,154],19:[326,154],20:[380,154],21:[434,154],22:[488,154],23:[603,154],24:[657,154],25:[711,154],26:[765,154],27:[886,154.66666666666669],28:[941.3333333333334,154.66666666666669],29:[996.6666666666667,154.66666666666669],30:[50,258],31:[104,258],32:[158,258],33:[212,258],34:[326,258],35:[380,258],36:[434,258],37:[488,258],38:[603,258],39:[657,258],40:[711,258],41:[765,258],42:[886,259.33333333333337],43:[941.3333333333334,259.33333333333337],44:[996.6666666666667,259.33333333333337],45:[50,362],46:[104,362],47:[158,362],48:[212,362],49:[326,362],50:[380,362],51:[434,362],52:[488,362],53:[603,362],54:[657,362],55:[711,362],56:[765,362],57:[886,364.00000000000006],58:[941.3333333333334,364.00000000000006],59:[996.6666666666667,364.00000000000006],60:[50,466],61:[104,466],62:[158,466],63:[212,466],64:[326,466],65:[380,466],66:[434,466],67:[488,466],68:[603,466],69:[657,466],70:[711,466],71:[765,466],72:[886,468.66666666666674],73:[941.3333333333334,468.66666666666674],74:[996.6666666666667,468.66666666666674],75:[50,570],76:[104,570],77:[158,570],78:[212,570],79:[326,570],80:[380,570],81:[434,570],82:[488,570],83:[603,570],84:[657,570],85:[711,570],86:[765,570],87:[886,573.3333333333334],88:[941.3333333333334,573.3333333333334],89:[996.6666666666667,573.3333333333334],90:[50,674],91:[104,674],92:[158,674],93:[212,674],94:[326,674],95:[380,674],96:[434,674],97:[488,674],98:[603,674],99:[657,674],100:[711,674],101:[765,674],102:[886,678],103:[941.3333333333334,678],104:[996.6666666666667,678],105:[50,778],106:[104,778],107:[158,778],108:[212,778],109:[326,778],110:[380,778],111:[434,778],112:[488,778],113:[603,778],114:[657,778],115:[711,778],116:[765,778],117:[886,782.6666666666666],118:[941.3333333333334,782.6666666666666],119:[996.6666666666667,782.6666666666666],120:[50,882],121:[104,882],122:[158,882],123:[212,882],124:[326,882],125:[380,882],126:[434,882],127:[488,882],128:[603,882],129:[657,882],130:[711,882],131:[765,882],132:[886,887.3333333333333],133:[941.3333333333334,887.3333333333333],134:[996.6666666666667,887.3333333333333],135:[50,986],136:[104,986],137:[158,986],138:[212,986],139:[326,986],140:[380,986],141:[434,986],142:[488,986],143:[603,986],144:[657,986],145:[711,986],146:[765,986],147:[886,991.9999999999999],148:[941.3333333333334,991.9999999999999],149:[996.6666666666667,991.9999999999999],150:[50,1090],151:[104,1090],152:[158,1090],153:[212,1090],154:[326,1090],155:[380,1090],156:[434,1090],157:[488,1090],158:[603,1090],159:[657,1090],160:[711,1090],161:[765,1090],162:[886,1096.6666666666665],163:[941.3333333333334,1096.6666666666665],164:[996.6666666666667,1096.6666666666665]}
